# Resolvin D1 attenuates the inflammatory process in mouse model of LPS‐induced keratitis

**DOI:** 10.1111/jcmm.15633

**Published:** 2020-10-15

**Authors:** Francesco Petrillo, Maria Consiglia Trotta, Claudio Bucolo, Anca Hermenean, Arianna Petrillo, Rosa Maisto, Gorizio Pieretti, Michela Pietropaolo, Franca Ferraraccio, Caterina Gagliano, Marilena Galdiero, Michele D'Amico

**Affiliations:** ^1^ Department of Ophthalmology University of Catania Catania Italy; ^2^ Department of Experimental Medicine University of Campania 'L.Vanvitelli' Naples Italy; ^3^ Department of Biomedical and Biotechnological Sciences School of Medicine University of Catania Catania Italy; ^4^ Institute of Life Science Vasile Goldis Western University Arad Romania; ^5^ Multidisciplinary Department of Surgical and Dental Specialties University of Campania'L. Vanvitelli' Naples Italy; ^6^ General Directorate of the University Polyclinic 'L. Vanvitelli' Naples Italy; ^7^ Department of Clinical, Public and Preventive Medicine University of Campania 'L. Vanvitelli' Naples Italy; ^8^ Eye Clinic University of Catania and Santa Marta Hospital Catania Italy

**Keywords:** cornea, Formyl peptide receptor 2, keratitis, lipopolysaccharide, Resolvin D1

## Abstract

The aim of this study was to investigate the effects of the lipid mediator Resolvin D1 in experimental keratitis. C57BL/6J mice were injected with lipopolysaccharide (2 µg/eye), and after 24 hours, the corneal damage was assessed. Clinical score was quantified, and corneal inflammatory biomarkers were detected by immunohistochemistry. A robust accumulation of sub‐epithelial macrophages and polymorphonuclear leucocytes, chemokine (C‐X‐C motif) ligand 1 (also known as keratinocyte‐derived chemokine), interleukin‐10 and promoters of apoptosis was also observed in lipopolysaccharide‐treated mice. Formyl peptide receptor 2 corneal expression was also assessed. The corneal stroma treated with lipopolysaccharide was characterized by presence of macrophages of M1‐like subtype and immature fibroblastic cells, marked with Ki67, not fully differentiated in fibroblasts. Indeed, the staining of the cornea with anti‐vimentin antibodies, a marker of differentiated myofibroblasts, was very faint. Resolvin D1 attenuated all the inflammatory parameters assessed in the present study, except for IL‐10. In conclusion, the data presented here seem to be consistent with the hypothesis that Resolvin D1 protected the cornea from the lipopolysaccharide‐induced keratitis by acting on several inflammatory components of this damage, pivoted by Formyl peptide receptor 2 (FPR2) activation and macrophages‐leucocytes activity.

## 1 INTRODUCTION

Keratitis is an ocular inflammatory disease characterized by corneal derangement, due to alterations of structural components such as tight junctions and infiltration of leucocytes into the cornea. These result in stromal keratitis, corneal thinning, and ultimately corneal perforation and scarring. Early neutrophil infiltration into the cornea causes local inflammation that greatly contributes to the host tissue destruction through the release of proteolytic enzymes and inflammatory mediators, ultimately leading to inflammation.^1^ This latter accompanied by damage of cellular components including cell membrane, cytoplasmic organelles and the nucleus, as for example the mitochondria and the DNA.

Several evidences recently highlighted the importance of a pro‐resolving novel strategy in the resolution of different experimental models of inflammation‐based pathologies.^2^, ^3^, ^4^, ^5^, ^6^, ^7^, ^8^ Among the specialized pro‐resolving mediators (SPMs), Resolvin D1 (RvD1) has emerged as a promising therapeutic tool. Derived from the w‐3‐polyunsaturated fatty acid (PUFA) docosahexaenoic acid (DHA),^9^ RvD1 was able to affect a pattern of mediators proper to inflammation and tissue repair, such as cytokines, IL‐10, keratinocyte‐derived chemokine (KC),^10^, ^11^ lymphocytes and M1/M2 macrophages^5^ in conjunctivitis,^12^, ^13^, ^14^ uveitis,^3^ choroid and retina,^7^, ^8^, ^15^, ^16^, ^17^, ^18^ by resolving several inflammation‐based ocular diseases.^3^, ^4^, ^5^, ^6^, ^15^, ^19^

Interestingly, RvD1 has shown anti‐inflammatory actions also in corneal epithelial cells, by inducing a dose‐response reduction of pro‐inflammatory cytokines and exerting positive actions on corneal epithelial wound healing.^20^, ^21^

However, despite these evidences, no study has explored, so far, the potential protective effect of RvD1 in keratitis. Therefore, a mouse model of lipopolysaccharide (LPS)‐induced keratitis has been to investigate the RvD1 actions, by monitoring the local inflammatory components such as KC, IL‐10 and macrophage phenotypes.

## 2 MATERIALS AND METHODS

### 2.1 Induction of keratitis and RvD1 treatment schedule

Keratitis was induced in male C57BL/6J mice (8‐10 weeks) by intrastromal injection of 2 μg LPS (*Pseudomonas aeruginosa*) (Sigma) dissolved in 2 μL of PBS (Sigma) in both the eyes. This LPS dose was previously reported as effective for the induction of keratitis in C57BL/6J mice.^22^ The intrastromal injection of RvD1 (Cayman Chemical) was carried out as mentioned before, and based on previous studies.^5^, ^6^ This compound was injected 60 minutes after LPS treatment, through the same tunnel created earlier, at the doses of 10‐, 100‐ and 1000 ng/eye/2 µL.

The mice were randomized in the following experimental group (n = 10 each): (a) vehicle group: mice receiving intrastromal injection of phosphate buffered saline (PBS; 2 µL); (b) LPS group: mice receiving intrastromal injection of lipopolysaccharide (LPS, 2 μg/eye/2 µL); (c) LPS + RvD1 10 group: mice receiving intrastromal injection of RvD1 10 ng/eye/2 µL before LPS; (d) LPS + RvD1 100 group: mice receiving intrastromal injection of RvD1 100 ng/eye/2 µL before LPS; and (e) LPS + RvD1 1000 group: mice receiving intrastromal injection of RvD1 1000 ng/eye/2 µL before LPS.

Twenty‐four hours after intrastromal injection, eyes were enucleated. For each experimental group, 10 dissected central corneas were placed in cooled PBS, isolated from other ocular tissues, immediately frozen in liquid nitrogen and stored at −80°C for subsequent biochemical analysis. The other 10 dissected central corneas were fixed by immersion in 10% neutral buffered formalin and paraffin‐embedded for immunohistochemistry.

Animal Ethics Committee of University of Campania'Luigi Vanvitelli'evaluated and approved the protocol (Protocol Number 2108), in accordance with Italian (DL 116/92) and European Commission (Directive 2010/63/EU) guidelines.

### 2.2 Clinical score

The development of keratitis was monitored 24 hours after LPS with a biomicroscope by an investigator unaware of treatments. Keratitis was scored as previously described,^1^ with some modifications. Briefly, the score was based upon the following criteria: 0 = normal, clear cornea, no inflammatory reaction; 1 = mild corneal haze with visible iris; 2 = moderate corneal flare with moderate corneal haze and superficial punctate keratitis; 3 = significant corneal opacity, infiltration of cells in the stroma; and 4 = damage and loss of corneal tissue. A score > 1 was assigned to positive keratitis.

### 2.3 Immunohistochemistry

Following cornea isolation, immunohistochemical analysis were performed as previously described by Rossi et al.^5^, ^6^ Briefly, paraffin was removed from paraffin‐embedded corneas by using a xylene substitute (Hemo‐De; Fisher Scientific); then, ethanol gradient washes were used in order to rehydrated cornea sections. These were quenched sequentially in 3% hydrogen peroxide aqueous solution, then blocked at room temperature for 1 hour with PBS 6% non‐fat dry milk (Bio‐Rad). Cornea sections were then incubated with the anti‐ataxia telangiectasia mutated (ATM) protein kinase (Mouse monoclonal anti‐ATM phospho S1981 antibody [10H11.E12]; dilution 1:1000; ab36810 Abcam), anti‐vimentin (Rabbit monoclonal anti‐vimentin antibody [EPR3776]; concentration 5 µg/mL; ab92547 Abcam), anti‐Ki67 (Rabbit polyclonal anti‐Ki67 antibody; concentration 5 µg/mL; ab155807 Abcam), anti‐integrin alpha X/CD11c antibody (Rabbit monoclonal anti‐CD11C antibody [EP‐134Y]; dilution 1:200; ab52632 Abcam) as marker for M1‐like macrophages, and anti‐mannose receptor antibody CD206 (Rabbit polyclonal anti‐mannose receptor [15‐2] antibody; concentration 5 µg/mL; ab64693 Abcam) as marker for M2‐like macrophages. The secondary antibodies used were biotin‐conjugated goat anti‐rabbit or antimouse IgG and avidin‐biotin peroxidase complex (Biotin goat anti‐rabbit IgG secondary antibody; dilution 1:1000; ab6720 Abcam and Biotin goat antimouse IgG secondary antibody; dilution 1:1000; ab6788 Abcam). A negative control was without primary antibody (data not shown). The immunohistochemical analysis was performed by an expert pathologist (intra‐observer variability 6%) unaware of the procedure. Antigen expression was measured and calculated automatically with the image program LEICA IMM500 and with the statistics program LEICA QWIN. In each experimental group, six distinct preparations were analysed by observing 20 microscopic fields, for a total area of 4.2575e + 005µm^2^ for 400× magnification.

### 2.4 Myeloperoxidase activity

The determination of myeloperoxidase activity was performed as described by D'Amico et al,^23^ Briefly, a snap‐frozen aliquot of the isolated cornea was homogenized in a buffer containing protease inhibitors (Sigma‐Aldrich) and centrifuged for 30 minutes at 4000 *g* at 4°C. Twenty μL of the supernatant was collected and then added to a solution of tetra‐methyl‐benzidine (1.6 mmol/L) and 0.1 mmol/L H_2_O_2_. A spectrophotometer set to 620 nm was used to analyse the change in absorbance for each sample.

### 2.5 Isolation and quantization of cornea protein content

Cornea tissues were homogenized in RIPA lysis buffer (Sigma; R0278) containing a protease inhibitor cocktail (Roche; 11873580001). Then, they were centrifuged at 13 000 *g* for 10 minutes at 4°C, to isolate nucleic acids from the protein supernatants. Bio‐Rad protein assay protocol (Bio‐Rad Laboratories; 500‐0006) was used to asses total protein concentration, used for ELISAs.

### 2.6 FPR2, RvD1, Connexin 43, cytokines, LY6G and p53 quantization by ELISA

Fifty μL of cornea homogenate was used to assess the protein levels of FPR2 (Myobiosource, MBS764510), RvD1 (Mybiosource, MBS058806), connexin 43 (Cnx43) (Myobiosource, MBS729401), chemokine (C‐X‐C motif) ligand 1 also known as keratinocyte‐derived chemokine (CXCL1/KC) (R&D Systems, MKC000B), interleukin‐10 (IL‐10) (R&D Systems, M1000B), lymphocyte antigen 6 complex locus G5C (LY6G5C) as marker of neutrophils (Mybiosource, MBS9339474) and p53 (Myobiosource, MBS721665). These were determined by using specific ELISA kits according to the manufacturer's instructions.

### 2.7 Statistical analysis

The results of each experiment were reported as the mean ± s.e.m. of n = 10 mice. One‐way ANOVA followed by Bonferroni's test was used to assess statistical significance among the groups with GraphPad Prism 6 software. A probability of *P* < .05 was considered sufficient to reject the null hypothesis.

## 3 RESULTS

### 3.1 Clinical score

**Figure 1:**
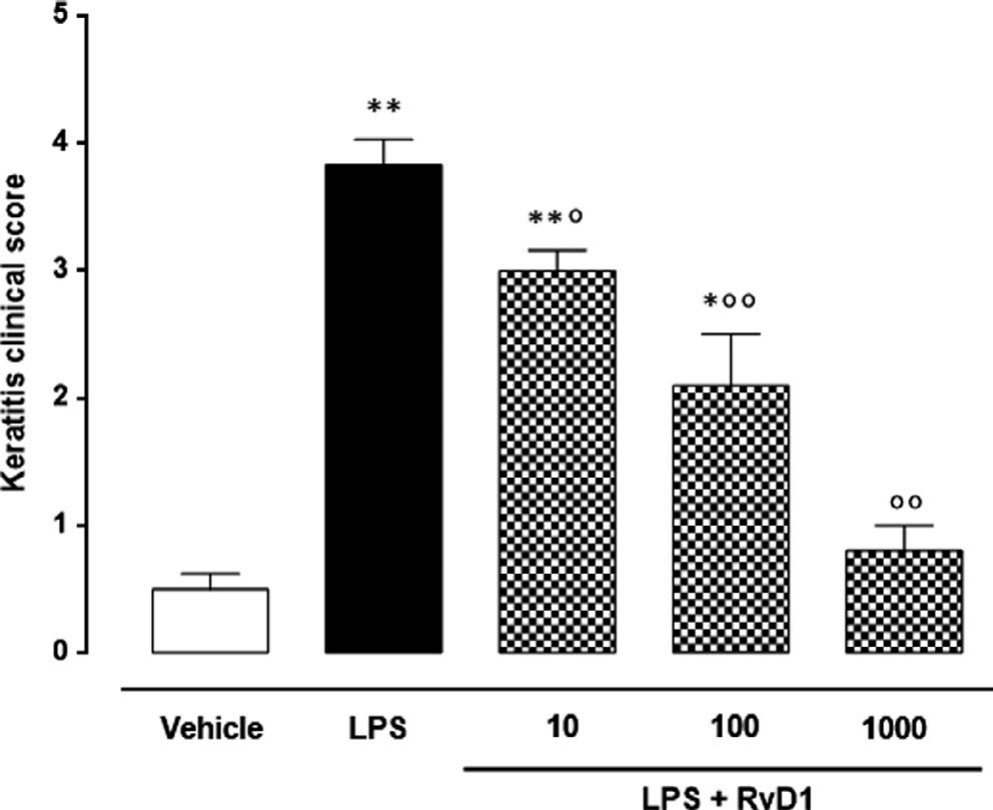
Clinical score of keratitis in vehicle (PBS), LPS (2 μg/eye) and LPS + RvD1 at the dose of 10‐, 100‐ and 1000 ng/eye groups. Values are reported as the mean ± s.e.m., of n* = *10 observations for each experimental group. **P < .01 vs vehicle; °*P* < .05 and °°*P* < .01 vs LPS

All the mice showed signs of keratitis 24 hours after LPS (2 µg/eye) (Figure 1), and severe alterations were evident in corneal structure (clinical score, 3.83 ± 0.2) (Figure 1).** **In contrast, intrastromal RvD1 injection (at doses of 10‐, 100‐, 1000 ng/eye) attenuated the inflammation (Figure 1), as showed by clinical score, elicited by LPS in a dose‐dependent manner (Figure 1). The maximum protection was reached at the dose of 1000 ng/eye, even though the other two (10 and 100 ng/eye) were significantly effective. Interestingly, because of the appearance of a phenomenon of bilateral ptosis observed in mice upon awakening from anaesthesia following the use of the 1000 ng RvD1 dose, the dose of 100 ng/eye was chosen the following biochemical and histological evaluations.

### 3.2 Haematoxylin/eosin stained cornea

**Figure 2:**
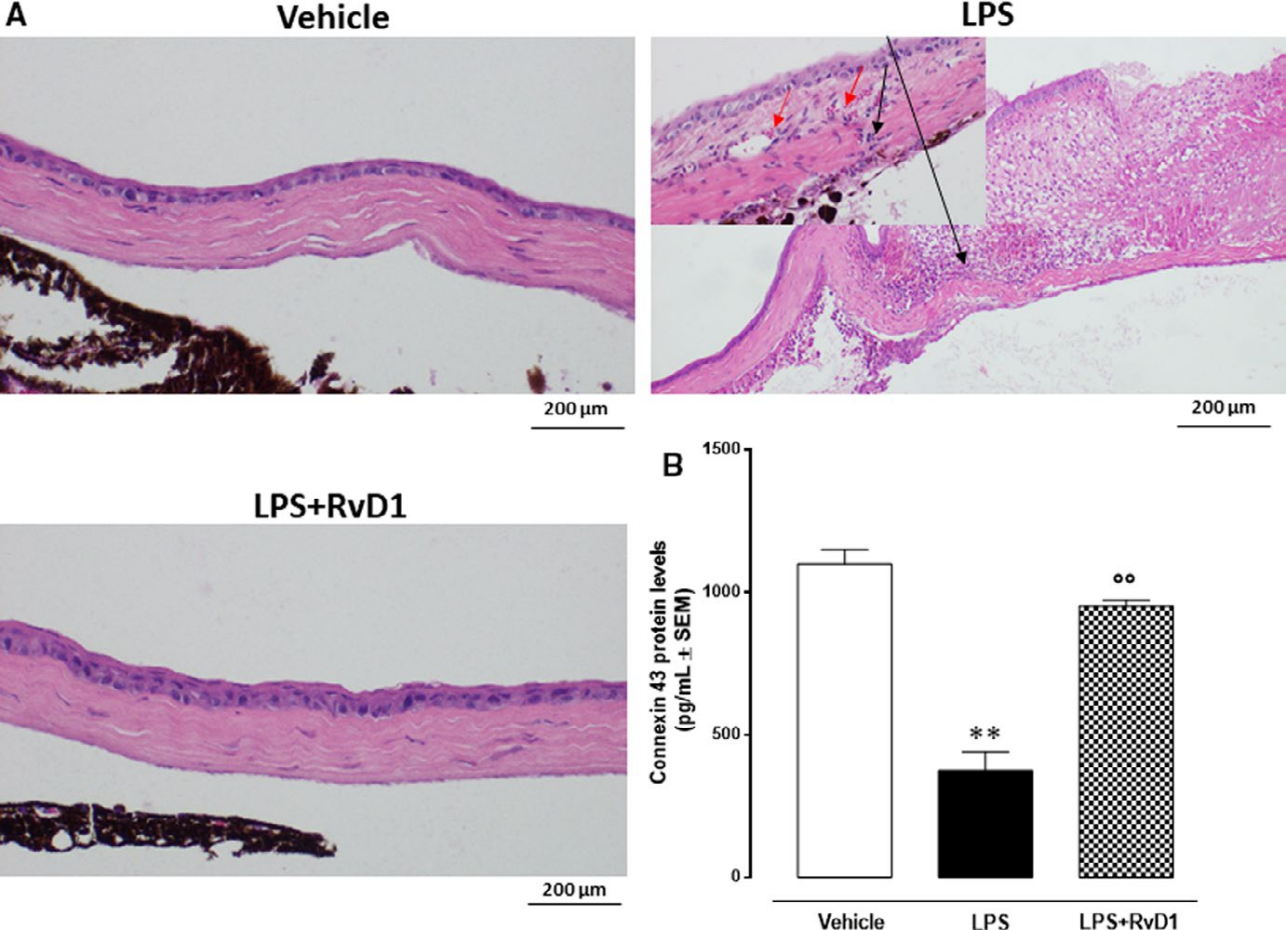
A, Haematoxylin/eosin staining of the cornea shows epithelial damage following LPS keratitis and protection from RvD1. Note the representative treatment with RvD1 (100 ng/eye) characterized by normal tissue structure in contrast to the LPS treated eye with normal epithelium on the left of the figure, on the right erosion of the epithelium with marked sub‐epithelial inflammatory infiltrate (black arrows) (20× magnification; scale bar = 200 μm). Greater magnification (40×), with associated oedema and erythrocytes extravasation (red arrow). B, Corneal Connexin 43 levels detected by ELISA in mice treated with vehicle (PBS), LPS (2 μg/eye) and LPS + RvD1 (100 ng/eye). Values are mean ± s.e.m. of n = 10 observations for group. **P < .01 vs vehicle; °°*P* < .01 vs LPS

Vehicle (PBS only) corneas showed normal epithelial cell and stromal morphologies. Corneas treated with LPS (2 μg/eye) had severe damage and showed a loss of cellularity of the basal and wing epithelial cells, flattening of the stromal lamellae and erosion of the epithelium compared to corneas of vehicle control mice (red arrows, Figure 2A). Sub‐epithelial inflammatory infiltrate, valuable by greater magnification, was observed with associated oedema and erythrocyte extravasation (black arrows, Figure 2A).

### 3.3 Connexin 43 in the corneal epithelium

Immunohistochemistry analysis evidenced a significant reduction of Cnx43 in the corneal epithelium in mice exposed to LPS (2 μg/eye) (−66%, *P* < .01 vs vehicle) (Figure 2B). Treatment of mice eyes with LPS + RvD1 (100 ng/eye) leads to a consistent restored expression of Cnx43 (+95.7%, *P* < .01 vs LPS) (Figure 2B).

### 3.4 Corneal FPR2 receptor, RvD1, LY6G expression and MPO activity

**Figure 3:**
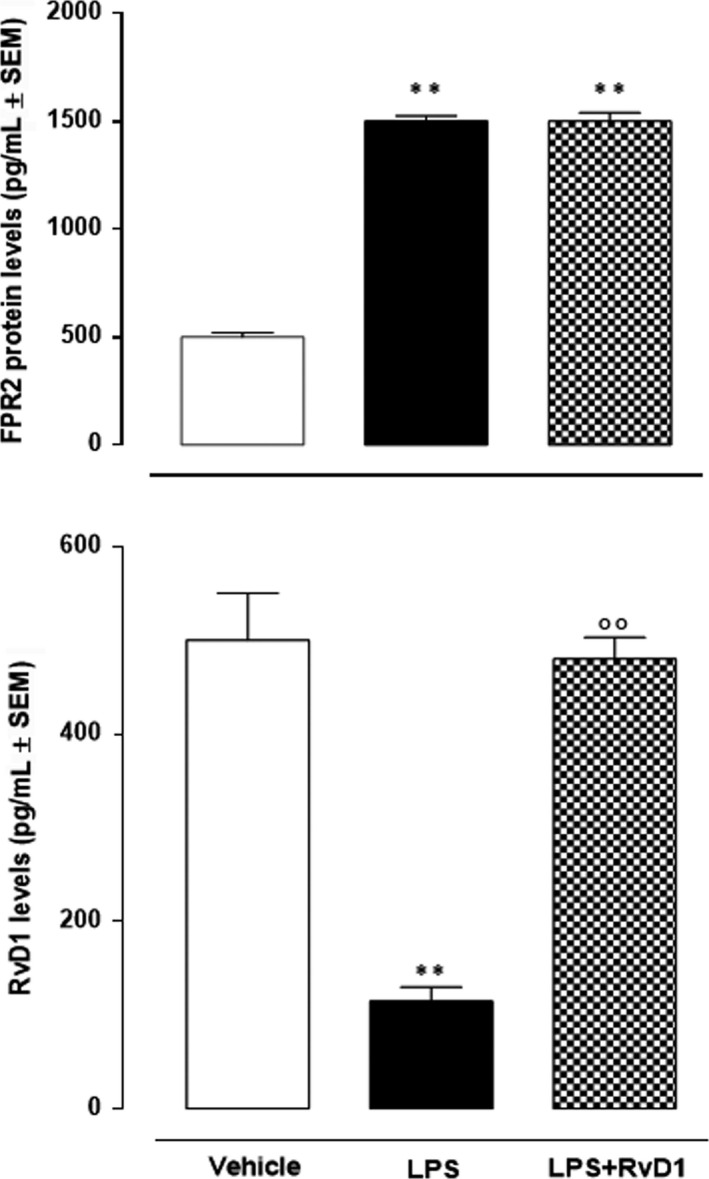
Endogenous levels of Resolvin D1 and formyl peptide receptor 2 (FPR2) expression in the cornea of mice treated with vehicle (PBS), LPS (2 μg/eye) and LPS + RvD1 (100 μg/eye), assessed by ELISA assay. Values are mean ± s.e.m. of n = 10 observations for group. **P < .01 vs vehicle; °°*P* < .01 vs LPS

Keratitis induced high expression of FPR2 receptor (3‐fold change vs vehicle) in the corneal homogenates. It was not affected by the treatment with RvD1 (Figure 3). In contrast, the endogenous levels of corneal RvD1 were low in LPS mice (−77%, *P* < .01 vs vehicle) and were markedly increased by exogenous RvD1 (+100%, *P* < .01 vs LPS;) (Figure 3).

**Figure 4:**
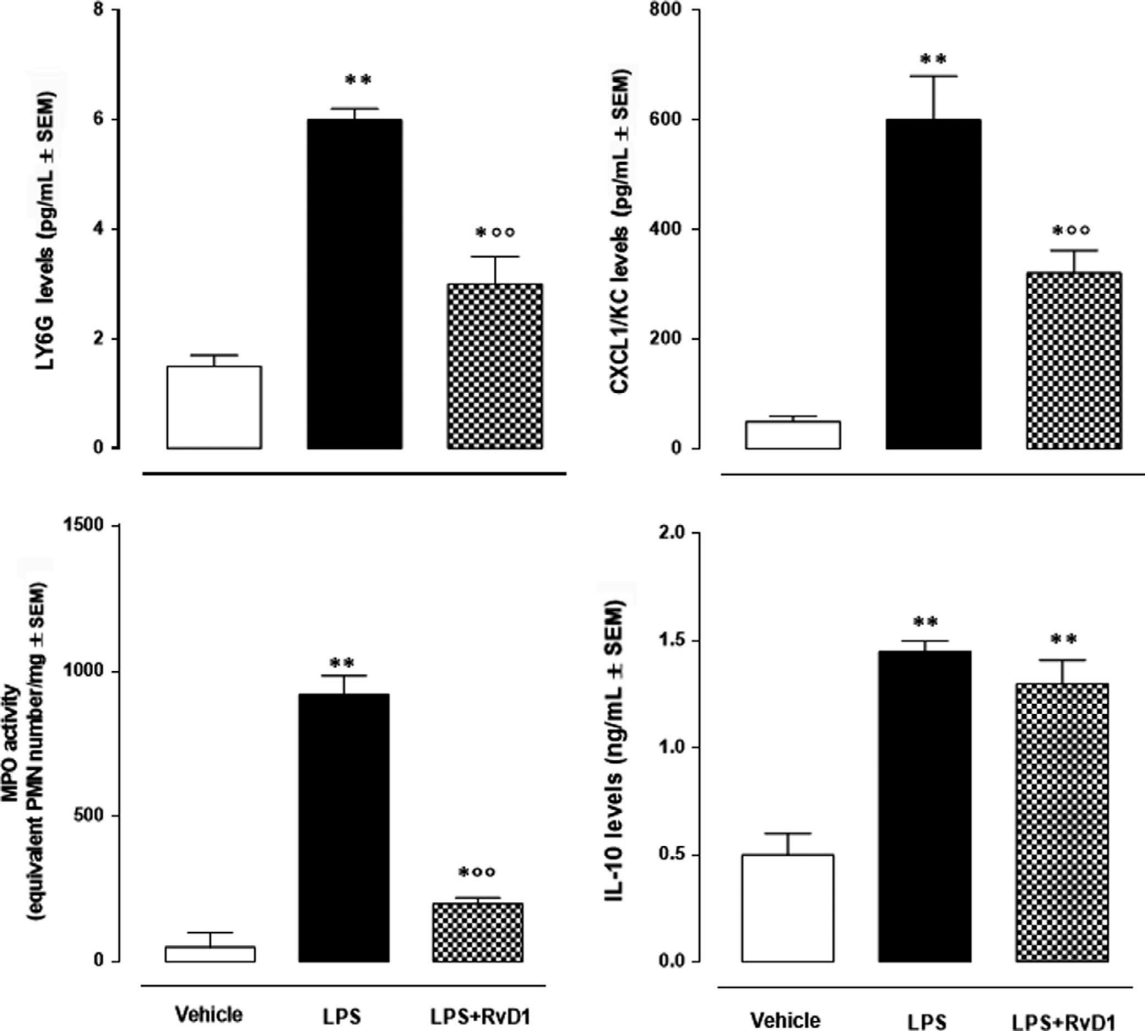
Levels of corneal LY6G (assessed by ELISA) and myeloperoxydase (MPO) activity in the cornea of mice treated with vehicle (PBS), LPS (2 μg/eye), LPS + RvD1 (100 ng/eye). ELISAs for CXCL1/KC and IL‐10 cytokines in the corneal homogenates of vehicle (PBS), LPS (2 ng/eye) and LPS + RvD1 (100 ng/eye)‐treated mice. Values are mean ± s.e.m. of n = 10 mice per group. *P < .05 and **P < .01 vs vehicle; °°*P* < .01 vs LPS

Paralleling the FPR2 expression, corneal sections assayed for LY6G in LPS mice showed highest content of this polymorphonuclear (PMN) leucocytes marker (*P* < .01 vs vehicle). In contrast, the RvD1 at dose of 100 ng/eye determined a decrease of this leucocyte marker with a significance being *P* < .01 with respect to LPS (Figure 4). This LY6G trend was copied by the MPO activity; thus, underlying PMN leucocytes increase into the corneal structures following LPS treatment and consequent decrease of it following RvD1 exposure (Figure 4).

### 3.5 RvD1 affected CXCL1/KC and IL‐10 cytokines

As per mirror of the biomolecular changes occurring after leucocytes infiltration, specific ELISA kits revealed a marked increase of both the PMN‐produced cytokine CXCL1/KC and the macrophage‐produced IL‐10 24 hours after the induction of keratitis (Figure 4). CXCL1/KC but not IL‐10 was affected by RvD1, indicating a major role for PMN activation rather than for macrophages or a possible shift of this leucocyte line towards the anti‐inflammatory phenotype (Figure 4).

### 3.6 RvD1 reduced the ATM and p53 markers

**Figure 5:**
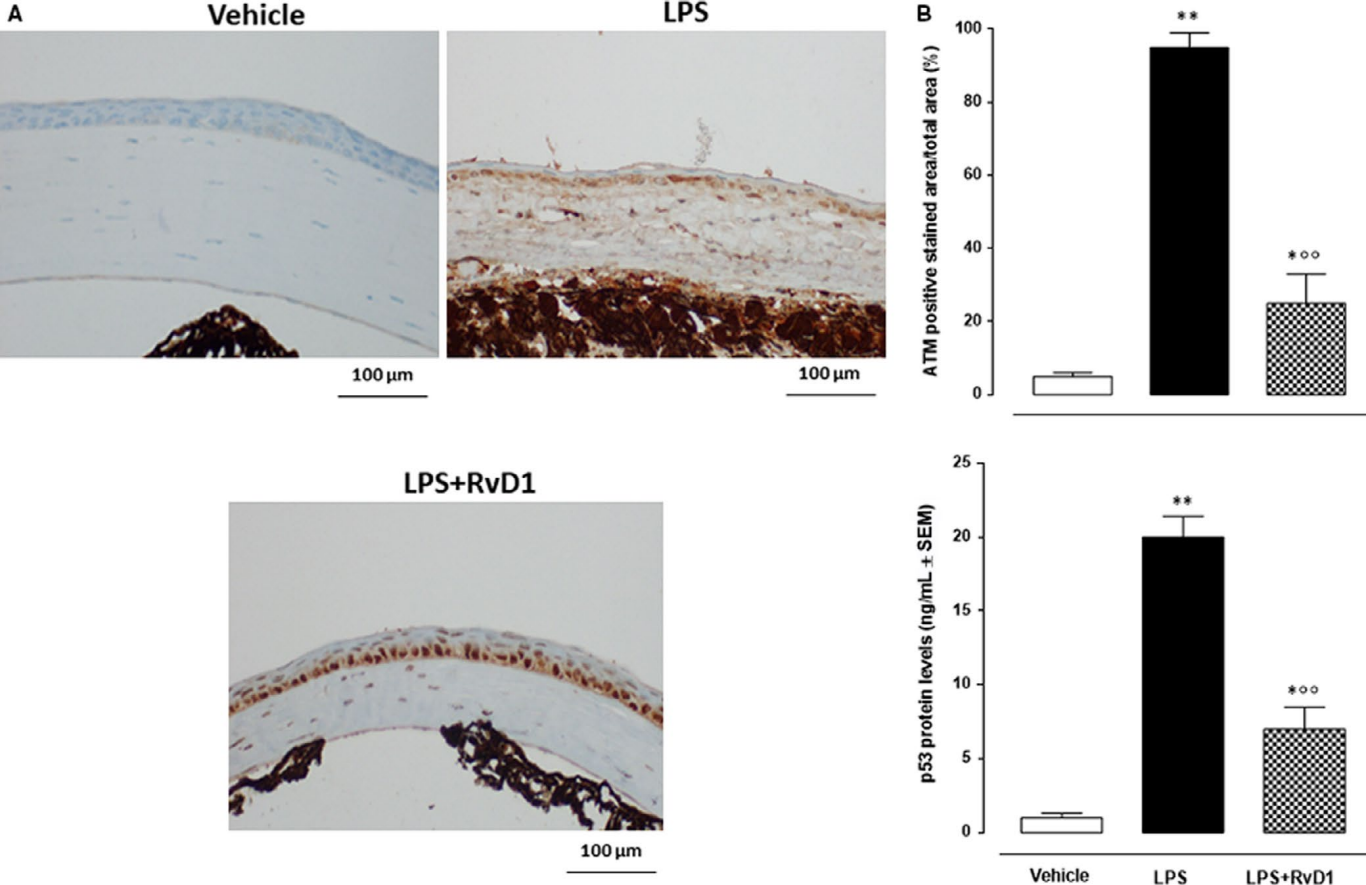
A, Representative immunohistochemistries (40× magnification; scale bar = 100 μm) showing ATM labelling in mice treated with vehicle (PBS), LPS (2 μg/eye) and LPS + RvD1 (100 ng/eye). B, Graphs showing the percentage of the ATM positive staining per total area analysed and p53 levels assayed by ELISA. Values are mean ± s.e.m. of n = 10 observation for each group. *P < .05 and **P < .01 vs vehicle; °°*P* < .01 vs LPS

Vehicle‐treated mice did not show any sign of apoptosis within the epithelium, stoma and basal membrane of the cornea (Figure 5). LPS significantly (+80%, *P* < .01 vs vehicle) increased the levels of the ATM serine/threonine kinase, sensor of DNA damage, and of the protein p53 (+73%, *P* < .01 vs vehicle) within the corneas of mice (Figure 5). A total of 100 ng/eye RvD1 decreased the ATM protein (−59.3%, *P* < .01 vs LPS) and the p53 protein (−62%, *P* < .01 vs LPS) (Figure 5).

### 3.7 Ki67 and vimentin

**Figure 6:**
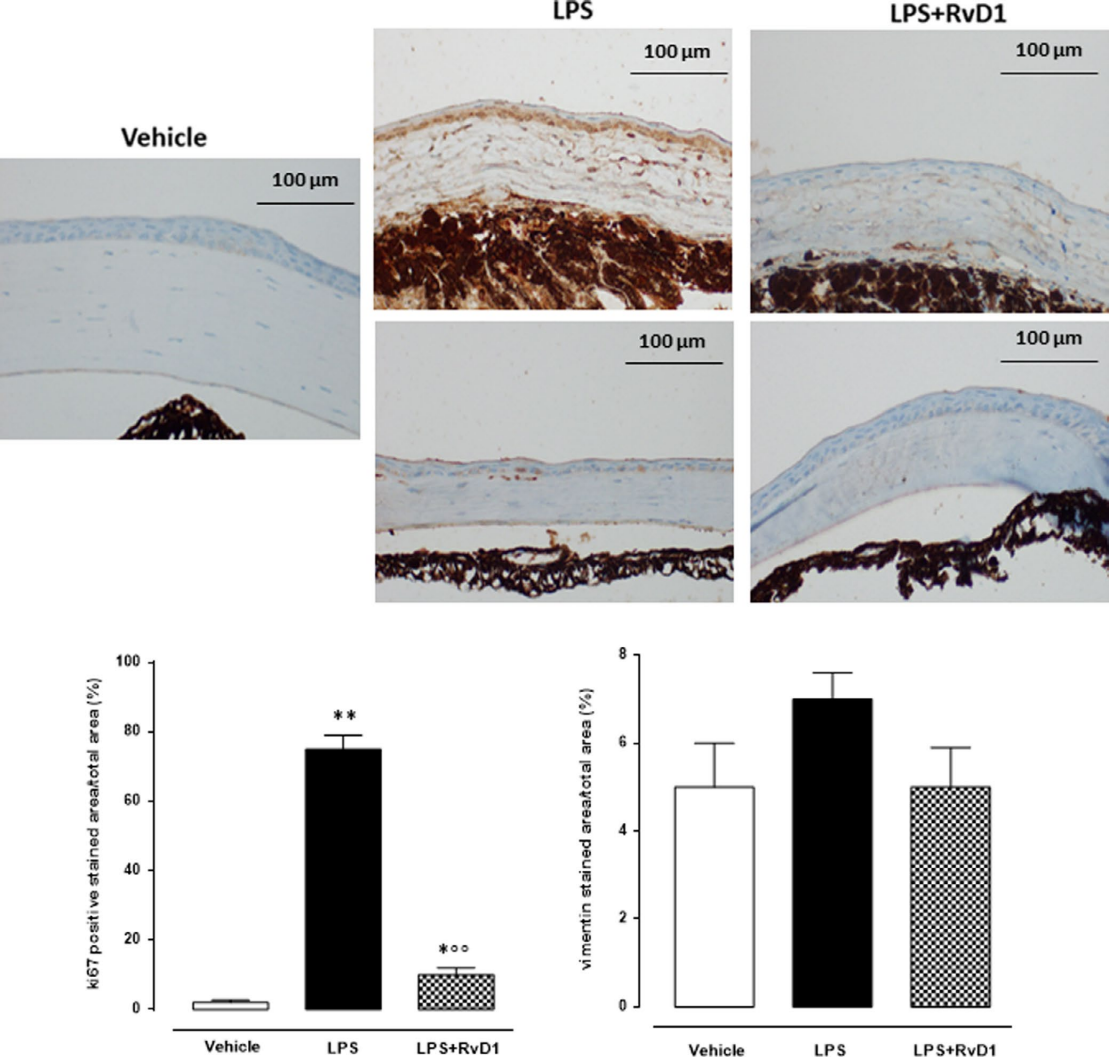
Ki67, marker of undifferentiated fibroblast into the cornea, was strongly reduced by RvD1. Vimentin (Vim), marker of differentiated fibroblasts, was low or absent in both LPS (2 μg/eye) and LPS + RvD1 (100 ng/eye) treated mice. Treatments as above. Graph showing the percentage of the total positive stained area per total area analysed at 40× magnification (scale bar = 100 μm). Values are mean ± s.e.m. of n = 10 observation for each group. *P < .05 and **P < .01 vs vehicle; °°*P* < .01 vs LPS

Ki67 as marker of undifferentiated stromal fibroblasts was evident after 24 hours of LPS compared to vehicle‐treated corneas and was reduced by 100 ng/eye RvD1 (Figure 6). Vimentin staining as marker of differentiated fibroblasts was faint in LPS‐corneas (Figure 6) with no significant difference with respect to vehicle corneas and with the groups treated with RvD1 (Figure 6).

### 3.8 Macrophage phenotypes were changed by intrastromal RvD1

**Figure 7:**
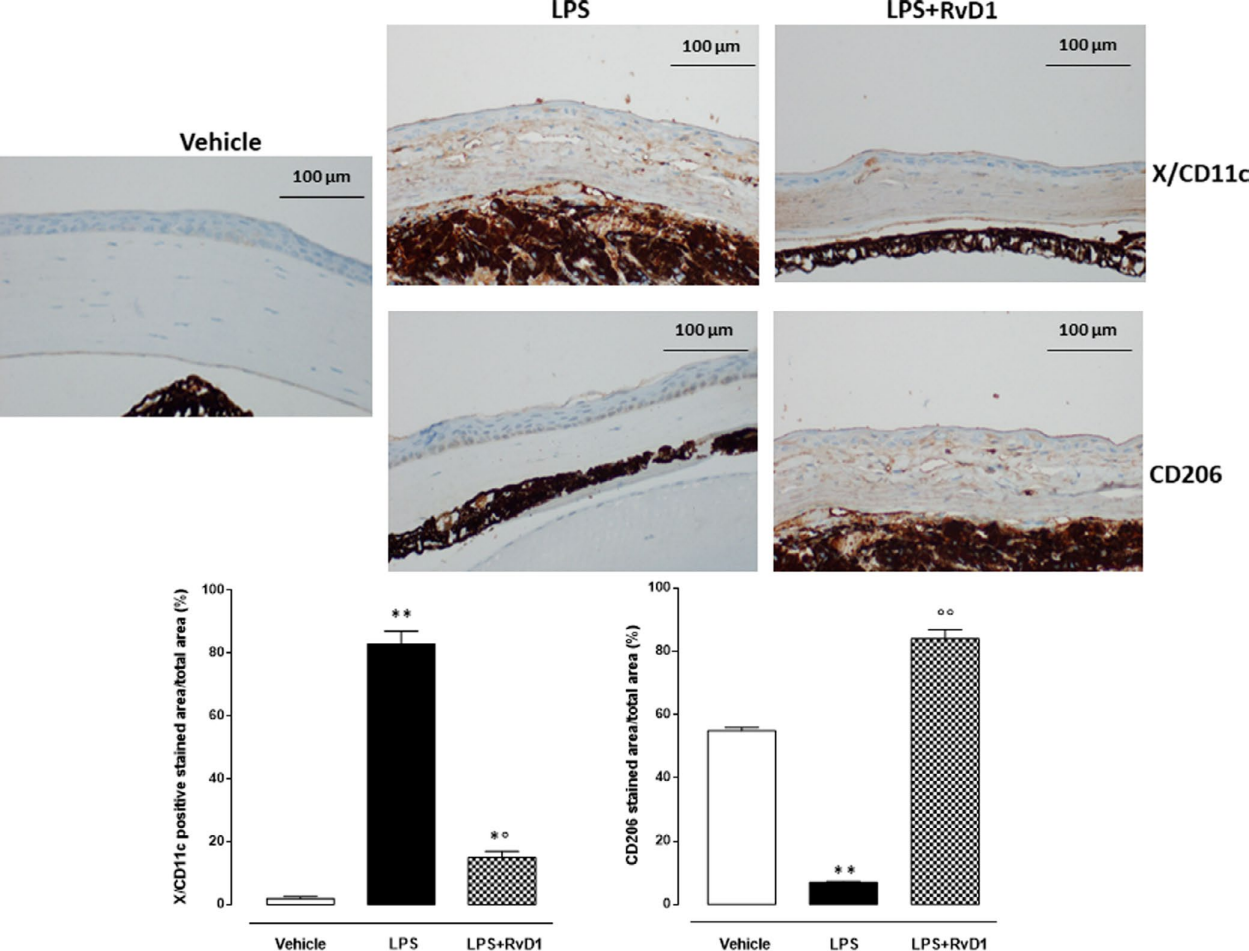
Anti‐CD11c and anti‐CD206 representative immunohistochemistries of cornea in both LPS (2 μg/eye) and LPS + RvD1 (100 ng/eye) treated mice. Graph showing the percentage of positive stained area per total area analysed at 40× magnification (scale bar = 100 μm). Values are mean ± s.e.m. of n = 10 observations for group. *P < .05 and **P < .01 vs vehicle; °°*P* < .01 vs LPS

Twenty‐four hours after the induction of keratitis, the corneas of the mice treated with LPS had a strong increase of the X/CD11c, marker of M1‐like macrophage phenotype, expression (Figure 7). The RvD1 (100 ng/eye 1 h post‐LPS) strongly reduced the increase of X/CD11c induced by LPS (Figure 7). In contrast to this, the expression of the macrophage M2‐like marker CD206 within the corneal structures was very low in LPS‐corneas with respect to the vehicle corneas, being the highest in the LPS + RvD1‐corneas (Figure 7).

## 4 DISCUSSION

In the present study, we showed that RvD1 ameliorated the pathological profile of LPS‐induced keratitis in mice. RvD1 reduced the corneal damage by affecting the neutrophil infiltration, the local release of CXCL1/KC, the LPS‐induced markers ATM and p53, the polarization of macrophages from M1‐like to M2‐like phenotype and reduced the proliferation of stromal fibroblast.

RvD1 is a specialized pro‐resolving mediator that exhibits anti‐inflammation, tissue protection and healing activities.^9^, ^21^, ^22^ It regulates neutrophil infiltration, alternative activated macrophage transition and pro‐inflammatory cytokine production by binding to formyl peptide receptor 2 (FPR2). This G protein‐coupled receptor is widely expressed by circulating blood neutrophils, eosinophils, basophils and monocytes; lymphocyte T cells and B cells; tissue Mast cells, macrophages, fibroblasts and immature dendritic cells; vascular endothelial cells; neural tissue glial cells, astrocytes and neuroblastoma cells; liver hepatocytes; and various types of epithelial cells.^24^, ^25^, ^26^, ^27^, ^28^ Once activated by RvD1, this receptor translates positive bioactive activities. FPR2 receptor is constitutively expressed in the cornea, as evidenced in the present study, and it is overexpressed in the cornea following LPS compared to vehicle cornea. In addition, this FPR2 overexpression is accompanied by low corneal endogenous RvD1 levels, data herein described for the first time. The current results reveal that these low levels are insufficient to fully activate the local overexpressed FPR2 receptors as their overexpression resulted in clear association with corneal damage in the LPS mice in our setting. Therefore, a strategic increase in RvD1 bioavailability and proper activation of FPR2 might counteract the corneal damage. In line with this contention, here it is demonstrated that an intrastromal administration of RvD1 increases corneal levels of RvD1 and was linked to reduction of LPS‐inflammation of the cornea through real FPR2 activation.

Among the biological activities of FPR2 receptor, there is the capability to limit the trafficking of neutrophils in the inflammation site.^29^, ^30^ Here, it is shown that exogenous RvD1, and proper activation of FPR2, promotes the diminution of PMN (neutrophils) presence in the corneal stroma. Although it is not possible to exclude the participation of other cells expressing FPR2 receptor in the action of RvD1, PMN trafficking seems to be one of the first component affected by RvD1 in the present setting of keratitis.

The corneal damage is mediated by autocrine and paracrine interactions of cytokines, growth factors, chemokines and their receptors produced by epithelial, stromal, bone marrow‐derived and neural cells that contribute to the cell damage.^31^ We demonstrated an increase of the IL‐8/CXCL8 homologue CCXL1/KC, probably generated by PMN leucocytes attracted into the corneal epithelial structures that may trigger the damaging phenomenon.^32^

Usually, the first and earliest damaging events start few hours after an inflammatory insult to the cornea^33^, ^34^, ^35^ and are followed by corneal cells necrosis mainly characterized by loss membrane integrity and random DNA degradation and is often associated with greater tissue injury and inflammatory response.^36^, ^37^ Here, we report that the marker of DNA breaking ATM serine/threonine kinase (ATM) is strongly augmented after LPS injection into the central stroma of the cornea, being reduced by the treatment with intrastromal injection of RvD1. ATM is a primary sensor protein of DNA double‐strand break (DSB) during cell cycle which signals post‐translational modifications to different mediators, such as Mre11 and MDC1, transducing the signals to downstream effectors such as p53, with the end‐point being cells damage.^38^

Following a stromal cells damage, there is often a proliferation and migration of residual activated keratocytes in the peripheral and posterior stroma within 12‐24 hours after corneal epithelial injury, aimed at repopulate the depleted stroma by means of immature and later fully differentiated myofibroblasts.^39^, ^40^, ^41^, ^42^, ^43^, ^44^ These latter cells are specialized fibroblastic cells generated in the cornea from keratocyte having a disorganized extracellular matrix components secreted that result in corneal stromal fibrosis that can compromise the clearness of the cornea. This migration and proliferation well evidenced by immunohistochemistry by using the antibody anti‐Ki67, a marker of cells undergoing mitosis. Here, it is shown that 24 hours after the induction of LPS keratitis there is a significant increase of the Ki67 binding within the stroma indicating a putative proliferation of cells surrounding the area of keratocyte apoptosis and necrosis. A plausible explanation of this phenomenon may reside in the migration of bone marrow‐derived cells into the stroma from the limbal blood vessels,^34^, ^45^ although more appropriate investigations are needed. Interestingly, the RvD1 decreased the corneal levels of the marker Ki67 expressed into the cornea, underlying a protective action against the proliferation of corneal keratocytes towards fibrosis. Real fibrosis, however, was not evident at this stage as the immunostaining for vimentin was bland. This is due to the fact that this protein, together with keratin, although one of the components of the intermediate filament produced by corneal epithelial cells during fibrosis, is transiently expressed in vivo during the early stages (first 24 hours) of epithelial damage and repair,^46^ thus justifying the absence observed in this study during the short acute period.

Last but not least is the action of RvD1 on corneal cytokines and chemokines release induced by LPS. The RvD1 decreased the levels of the chemokine CXCL1/KC within the cornea, as previously mentioned.

In contrast to this, RvD1 did not significantly affect the levels of the cytokine IL‐10. It should be noted that RvD1 may not influence IL‐10 levels as much as it would allow a macrophage shift from M1‐like to M2‐like phenotype that would support the resolution of the deleterious migration of neutrophils and degradation factors in the cornea. Indeed, the levels of M2‐like macrophages after RvD1 treatment were higher than the M1‐like, consistent with the high levels of IL‐10 within the cornea.

## 5 CONCLUSIONS

The results seem to be consistent with the hypothesis that RvD1 protected the cornea from the LPS‐induced keratitis by acting on several inflammatory components of this damage, pivoted by FPR2 activation and macrophages‐leucocytes activity. A mention is due to the appearance of a phenomenon of bilateral ptosis observed in mice upon awakening from the use of RvD1 1000 ng/eye requiring deeper investigation.

## CONFLICT OF INTEREST

The authors confirm that there are no conflicts of interest.

## AUTHOR CONTRIBUTIONS

**Francesco Petrillo:** Investigation (lead). **Maria Consiglia Trotta:** Data curation (lead). **Claudio Bucolo:** Writing‐review & editing (equal). **Anca Hermenean:** Investigation (supporting). **Arianna Petrillo:** Data curation (supporting). **Rosa Maisto:** Investigation (supporting). **Gorizio Pieretti:** Investigation (supporting). **Michela Pietropaolo:** Writing‐review & editing (equal). **Franca Ferraraccio:** Investigation (supporting). **Caterina Gagliano:** Investigation (supporting). **Marilena Galdiero:** Writing‐original draft (supporting). **Michele D'Amico:** Writing‐original draft (lead).

## Data Availability

The authors confirm that the data supporting the findings of this study are available within the article.
